# Newborn screening for Gaucher disease in Japan

**DOI:** 10.1016/j.ymgmr.2022.100850

**Published:** 2022-02-18

**Authors:** Takaaki Sawada, Jun Kido, Keishin Sugawara, Shinichiro Yoshida, Shirou Matsumoto, Tomoyuki Shimazu, Yuki Matsushita, Takahito Inoue, Shinichi Hirose, Fumio Endo, Kimitoshi Nakamura

**Affiliations:** a Department of Pediatrics, Graduate School of Medical Sciences, Kumamoto University, Kumamoto, Japan; b Department of Pediatrics, Faculty of Life Sciences, Kumamoto University, Kumamoto, Japan; c KM Biologics Co., Ltd, Kumamoto, Japan; d Department of Pediatrics, National Hospital Organization Kumamoto Saishun Medical Center, Kumamoto, Japan.; e Department of Pediatrics, Clinical Research Institute, Kyushu Medical Center, National Hospital Organization, Fukuoka, Japan; f Department of Pediatrics, School of Medicine, Fukuoka University, Fukuoka, Japan; g Department of Pediatrics, Fukuoka University Chikushi Hospital, Fukuoka, Japan; h General Medical Research Center, School of Medicine, Fukuoka University, Fukuoka, Japan; i Kumamoto-Ezuko Medical Center for Disabled Children, Kumamoto, Japan

**Keywords:** Enzyme replacement therapy, Gaucher disease, Glucocerebrosidase, Newborn screening, Neuronopathic Gaucher disease

## Abstract

Gaucher disease (GD) is an autosomal recessive inborn metabolic disorder caused by a glucocerebrosidase (GCase) defect. GD is classified into three main types depending on accompanying neurological symptoms. Enzyme replacement therapy and substrate reduction therapy are limited in the treatment of neurological symptoms, and using genotype and GCase activity to discriminate between non-neuronopathic and neuronopathic GD may be challenging as the two sometimes phenotypically overlap. The number of patients exhibiting neurological symptoms in Japan is significantly higher than that in Europe and the United States, and newborn screening (NBS) is still not actively performed in Japan. Definitive determination of the actual frequency and proportion of the type of GD from the results of NBS remains inconclusive. We performed NBS for Fabry disease, Pompe disease, and GD, mainly in the Kyushu area in Japan. Herein, we discuss the results of NBS for GD, as well as, the insights gained from following the clinical course of patients diagnosed through NBS. A total of 155,442 newborns were screened using an enzyme activity assay using dried blood spots. We found four newborns showing lower GCase activity and were definitively diagnosed with GD by *GBA* gene analysis. The frequency of GD diagnosis through NBS was 1 in 77,720 when limited to the probands. This frequency is higher than that previously estimated in Japan. In the future, NBS for GD is expected to be performed in many regions of Japan and contribute to detecting more patients with GD. Early screening and diagnosis may have a very significant impact on the quality of life and potentially longevity in infants with GD.

## Highlights

• Newborn screening (NBS) identified 4 cases of Gaucher disease (GD) with few false positives in Japan.
• The frequency of GD diagnosis through NBS was 1 in 77,720, being higher than the previously estimated.
• Early diagnosis may have a very significant impact on the quality of life and potentially longevity in infants with GD.

## 1 Introduction

Gaucher disease (GD) is an autosomal recessive inborn metabolic disorder caused by a defect in glucocerebrosidase (GCase; EC 3.2.1.45) in lysosomes [1]. It is caused by biallelic mutations in the *GBA* gene located on chromosome 1q21 [2]. GCase is mainly responsible for the breakdown of glucosylceramide into glucose and ceramide, and its dysfunction causes the progressive accumulation of glucosylceramide in the spleen, liver, bone marrow, bone, and other tissues and organs, resulting in anemia, thrombocytopenia, hepatosplenomegaly, and bone symptoms, such as bone pain, fractures, and avascular bone necrosis [3]. Accumulation of the deacylated form of glucosylceramide, glucosylsphingosine, in the brain is thought to result in the development of neurological symptoms in GD [4]. Definitive diagnosis of GD is accomplished by confirming a defect in GCase activity and/or by confirming biallelic pathological variants in the *GBA* gene [5,6].

GD can be classified into three main types depending on neurological symptoms [7,8]. Type 1 (GD1, OMIM 230800) is a non-neuronopathic form that can occur at various ages from infancy to adulthood. Type 2 (GD2, OMIM 230900) is an acute neuronopathic form that leads to death during infancy or early childhood. Type 3 (GD3, OMIM 23100) is a subacute or chronic neuronopathic form in which neurological symptoms develop later and progress slower than in GD2.

Treatment for GD includes enzyme replacement therapy (ERT), substrate reduction therapy (SRT), and hematopoietic stem cell transplantation (HSCT); early diagnosis is essential because treatment should be initiated before development of severe complications, such as massive fibrous splenomegaly, avascular necrosis, and pathological fractures [3]. The effect of these treatments on the neurological symptoms is insufficient; hence, the effectiveness of early detection of neuronopathic GD before the emergence of symptoms is controversial [9].

The prevalence of GD in the United States and Europe is estimated to be approximately 1 in 100,000 [10], and 90% of these patients suffer from GD1 [11]. According to a recent nationwide survey in Japan, the prevalence of GD is estimated to be 1 in 530,000, and more than 50% of these patients have GD 2 or GD3 [12]. However, the actual prevalence of GD in Japan may be higher than this estimated prevalence considering undiagnosed patients. Newborn screening (NBS) for GD is not encouraged or actively performed in Japan, and the definite prediction of the frequency and proportion of the type of GD from the results of NBS remains obscure.

NBS for Fabry disease [13] and Pompe disease [14] has been conducted at Kumamoto University since 2006 and 2013, respectively, and NBS for GD has been conducted since 2016. Herein, we present the results of NBS for GD at our institution conducted between 2016 and 2021. We also discuss the insights gained from following the clinical course of patients detected early through NBS.

## 2 Materials and methods

### 2.1 Study population

A total of 155,422 newborns were included in this study: 69,578 newborns from Kumamoto Prefecture from December 2016 to August 2021 and 85,844 newborns from Fukuoka Prefecture from April 2019 to August 2021. Informed consent was obtained from parents of the participating newborns and dried blood spot (DBS) samples were prepared in each maternity clinic or obstetric department using a heel-prick procedure 4–6 days after birth. The blood spots were blotted with filter paper (Toyo Roshi Kaisha, Ltd., Tokyo, Japan), the filter paper was dried for at least 4 h at room temperature (15–30 °C), and the samples were sent to the Newborn Screening Center at KM Biologics Co., Ltd. (Kumamoto, Japan) by mail, where public-funded NBS was conducted within 1 week of collection. The DBSs were then transferred to Kumamoto University to assay the GCase activity.

### 2.2 NBS program for GD

NBS for GD was performed in three steps. First, GCase activity was assayed using DBS samples. Newborns with GCase activity below the cutoff level (< 3 pmol/h/disk) were recalled, and DBSs were prepared again for the second GCase assay. The newborns whose GCase activity was still below the cutoff level were referred to the hospital for clinical examination, and physical examination and biochemical assays were performed to detect the symptoms of GD. Informed consent was obtained and the *GBA* gene in such newborns was sequenced for confirming the diagnosis.

### 2.3 GCase assay of the DBS

#### 2.3.1 Method I

Method I of GCase assay was developed in collaboration with KM Biologics Co., Ltd. (see details in JP6360848B) and implemented in December 2016. Briefly, disks (3.2 mm in diameter) were punched from the DBS cards and placed one each into each well of a 96-well clear microwell plate (Corning, NY, USA) with 100 μL of extraction solution (0.1% TritonX-100, 5 mM MgCl_2_, 0.5 mM dithiothreitol, and 0.05% NaN_3_ in 24 mM citrate potassium phosphate buffer, pH 6.0) for 1 h at room temperature (15–30 °C). In a 96-well black microwell-plate (PerkinElmer, Waltham, MA, USA), a 20 μL aliquot of the extract was then added to 40 μL of substrate solution (3 mM 4-methylumbelliferyl-β-D-glucopyranoside (4-MU-β-D-Glc) and 0.3% sodium taurodeoxycholate in 100 mM citrate sodium phosphate buffer, pH 5.0) and incubated at 38 °C for 3 h, after which 200 μL of reaction stop solution (300 mM glycine/NaOH buffer containing 10 mM EDTA, pH 10.6) was added. The fluorescence intensity was analyzed at 370 nm excitation and 465 nm emission wavelengths. Enzyme activity was expressed as picomoles of 4-methylumbelliferone (4-MU) released per hour per disk (pmol/h/disk).

#### 2.3.2 Method II

Method II for high-throughput assays was developed in collaboration with KM Biologics Co., Ltd. (see details at P2018-102295A) and implemented in February 2019. Briefly, the compositions of the extraction solution and substrate solution were modified as follows: extraction buffer (0.1% TritonX-100, 38 mM KCl, 5 mM MgCl2, 0.18 mM dithiothreitol, and 0.05% NaN3 in 5 mM sodium acetate buffer, pH 5.2) and substrate solution (5 mM 4-MU-β-D-Glc, 0.3% sodium taurodeoxycholate in 100 mM citrate sodium phosphate buffer, pH 5.0). The remaining protocol was the same as in method I.

#### 2.3.3 Method III

Method III was performed using the Enzaplate LSD assay kit (Siemens Healthcare Diagnostics K.K., Tokyo, Japan). The kit was developed by the Daiichi Kishimoto Clinical Laboratories (Sapporo, Japan) and Sapporo Immuno Diagnostic Laboratory Co., Ltd., using the intellectual property of P2017–245523 under the license of KM Biologics Co. Briefly, single 3.2 mm diameter disks punched from DBSs were incubated in a 96-well clear microwell-plate with 200 μL of extraction solution (included in the kit). In a 96-well black microwell-plate, a 20 μL aliquot of the extract was then added to 40 μL of substrate solution (included in the kit) and incubated at 38 °C for 3 h, after which 200 μL of reaction stop solution was added (included in the kit). The fluorescence intensity was analyzed at 370 nm excitation and 465 nm emission wavelengths. Molar product quantities in the assay wells were calculated from the linear regression of the standard curve. The enzyme activity was expressed as picomoles of 4-MU released per hour per disk (pmol/h/disk).

### 2.4 *GBA* gene analysis

*GBA* gene analysis was performed using long-range PCR and next-generation sequencing [15]. *GBA* and its flanking regions were amplified using forward primer 5′-TTGTCACACTGAACACATCCAGCC-3′ and reverse primer 5′-CAGAGACTGAGGAGGAGCCATACC-3′ (Supplemental data 1). Long-range PCR was optimized to amplify the *GBA* and flanking regions. PCR was performed using KOD FX (Toyobo, Osaka, Japan) as follows: 94 °C for 2 min; followed by 30 cycles at 98 °C for 10 s and 64.3 °C for 30 s; and 68 °C for 13 min 36 s using a Veriti Thermal Cycler (Applied Biosystems, Foster City, CA, USA). After checking by agarose gel electrophoresis, PCR products (amplicons) were purified using an Agencourt AMP XP PCR Purification Kit (Beckman Coulter, Brea, CA, USA) and quantified with a Qubit dsDNA HS Assay Kit (Life Technologies, Carlsbad, CA, USA) using a Qubit 2.0 Fluorometer (Life Technologies). Simultaneous fragmentation and adaptor ligation of PCR products were performed using a Nextera XT Kit (Illumina, San Diego, CA, USA). Indexed DNA was purified using the Agencourt AMP XP PCR Purification Kit. Each library was validated with High Sensitivity D1000 ScreenTape (Agilent Technologies, Santa Clara, CA, USA) using an Agilent 2200 TapeStation and quantified with a Qubit dsDNA HS Assay Kit using a Qubit 2.0 Fluorometer to allow for library normalization. Sequencing was performed with a MiSeq Reagent Kit v3, 150 cycles (Illumina) and MiSeq sequencer using the “paired-end” sequencing run method. Sequence data analysis, mapping, and variant calling were streamlined using MiSeq Reporter v2 (Illumina). Briefly, reads were aligned to the reference sequence, from 155,247,771 to 155,234,147 of the genome sequence of chromosome 1 (NC_000001.11), using bwa-0.6.1. Single-nucleotide polymorphism (SNP) and insertion/deletion (INDEL) identification was performed using the Genome analysis toolkit (GATK v1.6; Broad Institute, Cambridge, MA, USA). Visualization of sequencing reads was performed with IGV_2.3.40 (Broad Institute).

### 2.5 Ethics

This study was approved by the Ethics Committee of Kumamoto University (approval no. 1537). Written informed consent was obtained from parents or legal guardians of the newborns.

## 3 Results

### 3.1 NBS for GD

**Fig. 1:**
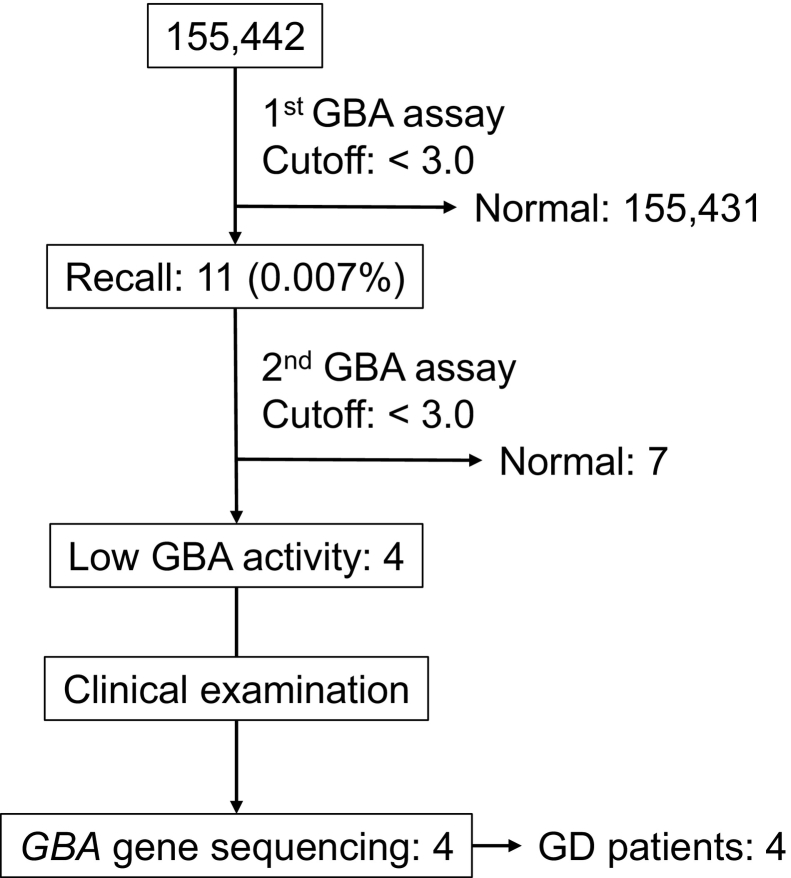
Flowchart of newborn screening for Gaucher disease.

**Fig. 2:**
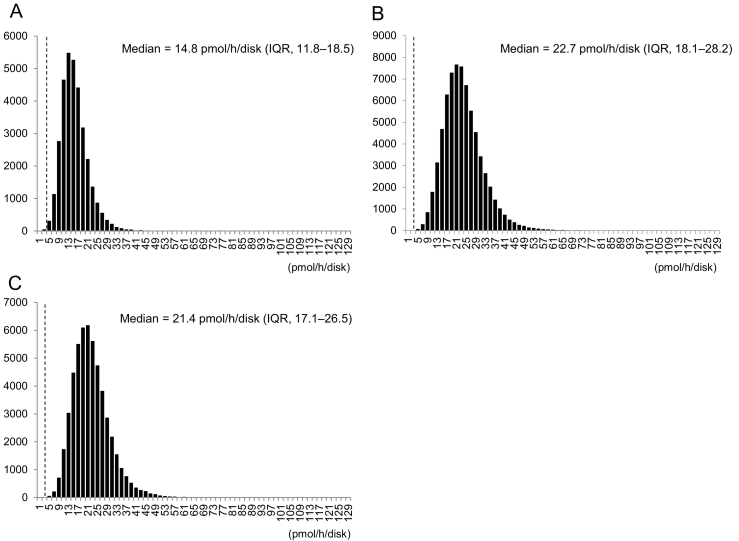
Histograms of glucocerebrosidase (GCase) activity in newborns. A. Method I (*N* = 33,508), Median GCase activity: 14.8 pmol/h/disk (IQR, 11.8–18.5). B. Method II (*N* = 69.307), Median GCase activity: 22.7 pmol/h/disk (IQR, 18.1–28.2). C. Method III (*N* = 52,627), Median GCase activity: 21.4 pmol/h/disk (IQR, 17.1–26.5). Dash line: cutoff level.

A flowchart and results of the NBS are presented in Fig. 1. A total of 155,442 newborns were screened for GCase activity using the DBSs. The median GCase activity was 14.8 pmol/h/disk (interquartile range [IQR]: 11.8–18.5) by Method I, 22.7 pmol/h/disk (IQR: 18.1–28.2) by Method II, and 21.4 pmol/h/disk (IQR: 17.1–26.5) by Method III (Fig. 2). Based on preliminary studies, we set the cutoff at 3.0 pmol/h/disk, which was 20% of the control. Eleven newborns (0.007%) were recalled, and finally, four showed impaired GCase activity during the second GCase activity measurement. The characteristics of the four patients are shown in Table 1. These four patients underwent physical examination, biochemical tests, imaging studies, and *GBA* gene analysis. All four patients who underwent *GBA* analysis were found to be homozygous or compound heterozygous for the pathogenic variants. Two of the four patients had a known family history of GD (Table 1). The frequency of patients with GD who did not present with a family history of GD at the time of the screening test (probands) was 1 in 77,720 (0.001%).

**Table 1:** Characteristics of Gaucher disease patients.

	Patient 1	Patient 2	Patient 3	Patient 4
Gender	Female	Male	Female	Male
Family history of GD at birth	No	Yes	No	Yes
Age at first examination	0 day	14 days	24 days	27 days
Initial examination				
Neurological findings	Yes	No	No	No
Hemoglobin level (g/dL)	15.2	13.6	11.4	11.7
Platelet count (×10^3/mL)	17	191	88	65
Hepatosplenomegaly	Yes	No	Yes	Yes
GCase activity in DBS (pmol/h/disk)	0.4	0.4	0.6	1.0
*GBA* gene variants	p.L483R	IVS2 + 1G > A	p.L483P	p.L483P
	p.L483R	p.M85T	p.L483P	p.L483P
Age at starting ERT	47 days	14 days	84 days	55 days
Age at last follow-up	Died at 2 months	2 years	2 years	1 year
Symptoms at last follow-up	Fixation of the eyeball, Laryngeal wheezing, Feeding disorder, Hypertonia	Strabismus, Hypertonia, Scoliosis, Psychomotor retardation	Slow horizontal saccades	None
Classification	Perinatal-lethal GD2	GD3	GD3	GD3⁎

GD: Gaucher disease, GCase: glucocerebrosidase, DBS: dried-blood spot, ERT: enzyme replacement therapy, GD2: type 2 Gaucher disease, GD3: type 3 Gaucher disease.

⁎ Estimated from genotype.

### 3.2 Patients detected in this study

We diagnosed four patients with GD through the NBS (Table 1). Patient 1 was a female infant born to Nepalese parents at 39 weeks' gestation. She was admitted to the neonatal intensive care unit immediately after birth because of respiratory failure. At birth, she developed collodion-like skin, hepatosplenomegaly, thrombocytopenia (17 × 10^3^/mL), hypertonia, and laryngeal wheezing. There were no findings of polyhydramnios, fetal hydrops, or impaired fetal movement during pregnancy. During NBS, her GCase activity was found to be decreased to 0.4 pmol/h/disk, and *GBA* gene analysis revealed that she carried the homozygous p.L483R (c.1448 T > G) variant. This variant has been reported in a patient with GD with compound heterozygous pathogenic variants [16]. She started receiving ERT from day 47 onwards, which eliminated the need for repeated platelet transfusions. She was discharged from the hospital at 57 days of age but died of asphyxia after vomiting at the age of 82 days. Based on her clinical course, we diagnosed her with perinatal-lethal GD [17,18].

Patient 2 had siblings diagnosed with GD2. During prenatal diagnosis, the patient was confirmed to have the same *GBA* variants as his siblings, but his mother continued the pregnancy to term and gave birth. During NBS, his GCase activity was found to be decreased to 0.4 pmol/h/disk. Postnatal *GBA* gene analysis revealed that he carried the compound heterozygous variants IVS2 + 1G > A (c.115 + 1G > A) and p.M85T (c.371 T > C). IVS2 + 1G > A causes a splice defect that skips exon 2 [19]. GD2 has been reported to occur when this variant is compound heterozygous with p.L483P or p.F290L [20]. p.M85T has been found in patients with GD [21] but no detailed clinical manifestations have been reported. The patient showed no symptoms of GD, including hematologic abnormalities, hepatosplenomegaly, or neurological symptoms, at the time of the initial examination on day 14 but ERT was initiated on the same day. He showed no hematological abnormalities, hepatosplenomegaly, or growth disorder, but strabismus, hypertonia, and scoliosis appeared around the age of two, and his psychomotor development was gradually delayed.

Patient 3 was the first child born to non-consanguineous parents. She presented to our institution at the age of 24 days with a GCase activity level of 0.6 mol/h/disk, which was below the cut-off value set in the NBS. On examination, lowered hemoglobin level (11.4 g/dL), decreased platelet count level (88 × 10^3^ /mL), and hepatosplenomegaly were noted. *GBA* analysis revealed that she was homozygous for the p.L483P (c.1448 T > C) variant. p.L483P is the most common pathogenic variant in Japanese patients with GD [22] and is involved in the development of neurological symptoms [23,24]. ERT was started at the age of 84 days and improved her anemia, thrombocytopenia, and hepatosplenomegaly. She developed slow horizontal saccades at the age of one year, but no other neurological symptoms appeared at the age of two years.

Patient 4 was the younger brother of Patient 3 and came to our institution at the age of 27 days with a low GCase activity level of 1.0 pmol/h/disk. On examination, lowered hemoglobin (11.7 g/dL) and platelet count (65 × 10^3^ /mL) levels, and hepatosplenomegaly were observed. No obvious neurological symptoms were noted. *GBA* analysis revealed that he was homozygous for the p.L483P variant. He showed improvement in hematologic abnormalities and hepatosplenomegaly after the initiation of ERT at age of 55 days. At the age of one year, he did not develop slow horizontal saccades.

## 4 Discussion

This NBS study for GD enrolled 155,442 newborns, of which four were diagnosed with GD. There are so far no patients developing GD that have been missed by our screening tests, although there is a possibility of obtaining a few false negative results.

The *GBA* gene comprises 12 exons and 11 introns spanning 10.2 kb. A highly homologous pseudogene (*GBAP1*) is located 17.1 kb downstream, with the same organization of exons and introns. Moreover, metaxin (*MTX1*) and its pseudogene *MTX1P1* are adjacent to *GBAP1* and *GBA*, respectively (Supplemental data 1A and B). The exonic sequence homology between *GBA* and *GBAP1* is more than 86%, increasing enhancing the likelihood of homologous recombination. Several different recombinant alleles and their crossover sites have been reported [[21], [25]] (Supplemental data 2). The presence of the highly homologous pseudogene makes analysis of the *GBA* gene challenging. Therefore, adequate strategies should be adopted to avoid diagnostic errors. The reverse primer used in this screening was designed to match the same sequence in *MTX1P1 and MTX1*. By using this primer set, a 13.6 kb amplicon containing *GBA* was obtained (Supplemental data 1). The other possible amplicon of 34.3 kb was too long for amplification. Since the recombination between *GBA* and *GBAP1* might shorten the length, recombinant allele amplifications is possible. If characteristic nucleotide substitutions and/or deletions are detected, it suggests that the subject has a recombinant allele. However, there is a limitation in our system. Because the difference in the sequence from intron 10 to exon 12 between *GBA* and *GBAP1* is small, it is difficult to determine whether c.1448 T > C (for example) is derived from Rec 1 or not.

The frequency of GD diagnosis through NBS was 1 in 38,861 when siblings were considered, and 1 in 77,720 when the screening was limited to probands. This frequency of GD is similar to that recently reported in NBS in Taiwan (1 in 73,743) [26] and China (1 in 80,844) [27]. Some patients diagnosed with GD included in these two studies were compound heterozygotes for p.L483P, which was also observed in our study. In contrast, the NBS in Illinois screened 219,937 newborns and detected 5 newborns with GD (1 in 43,959). Of these five newborns, four were homozygous for the p.N409S (c.1226A > G) variant [28]. Moreover, in the NBS of 65,605 newborns in New York, 15 (1 in 4374) were diagnosed with GD, and 14 of them were homozygous for the p.N409S variant [29]. All 14 patients diagnosed with GD were Ashkenazi Jewish, reflecting the high Ashkenazi Jewish population with the p.N409S variant in New York City. The p.N409S variant is associated with GD1 and is predominantly prevalent in Ashkenazi Jewish [30].

Infants with GD detected during our screening presented with varying neurological symptoms. Of these, patient 1 developed the most severe neurological symptoms, and we diagnosed her with perinatal-lethal GD [17,18]. She harbored the homozygous p.L483R variant. Her symptoms were more severe than those of GD patients homozygous for p. L483P, which is a mutation at the same site. This suggests that p.L483R is a more severe variant than p. L483P. No case of GD with the p.L483R variant has been reported in Nepal, and it is unclear whether p.L483R is a common variant in Nepalese. ERT has been approved as the first-line treatment for GD2 in Japan [31]. The first patient's hematological abnormalities and hepatosplenomegaly improved after ERT but her neurological symptoms gradually worsened. She was discharged from the hospital and was able to spend time with her parents at home.

Patient 2 had siblings who were diagnosed with GD2; therefore, he was screened for GD and expectedly developed neurological symptoms after GD diagnosis. One of his siblings was introduced to our institution with strabismus and abnormal eye movements at age of 11 months and was diagnosed with GD at the age of one year with thrombocytopenia and hepatosplenomegaly. His onset was later than that seen in typical GD2; however, the rapid progression of hypertonia, seizures, and dysphagia led to the diagnosis of GD2. Schiffmann et al. defined the neurological symptoms of GD2 as appearing within the age of 6 months [32] but cases in which neurological symptoms develop after 6 months and progress rapidly have also been diagnosed as GD2 [18]. Patient 2 was started on ERT before GD symptoms appeared, and he did not develop hematological abnormalities, hepatosplenomegaly, or growth disorder, as in his siblings, but strabismus, hypertonia, and psychomotor development delay appeared at approximately 2 years of age. His phenotype was similar to that in GD3a, characterized by predominant neurological involvement, and even siblings with the same genotype showed different phenotypes.

Patients 3 and 4 were siblings and were homozygous for p.L483P. This variant is the most frequent in patients with neuropathic GD [33] and Japanese patients with GD [22]. Although most patients with the homozygous p.L483P variant develop GD3 [24,30], they may have a variety of clinical courses [23]. There is an unclear relationship between the clinical course of GD and residual GCase activity [23]. Patient 3 showed slow horizontal saccades since the age of 1 year, whereas patient 4 had no horizontal saccades. In another high-risk screening for GD in patients with neurological symptoms, two children with eye movement disorders were diagnosed with GD [34]. In addition to eye movement disorders, ophthalmic manifestations, such as ocular muscle paralysis, nystagmus, and corneal opacity have been reported in GD [35]. Saccadic eye movements in patients with GD3 have been suggested to reflect neurological disorders [36]. This suggests that ophthalmologic examination should be considered in the evaluation of neurological symptoms in patients with GD. More definitive diagnostic criteria for neurological GD are needed in younger patients.

All patients detected during NBS in our study had no neurological symptoms at the start of treatment, except for Patient 1. However, ERT and SRT could not prevent the onset of or ameliorate neurological symptoms because the enzymes and compounds used in these therapies cannot pass through the BBB [37]. HSCT has been shown to be effective for neurological symptoms in some cases but the evidence is insufficient [38]. Currently, medical therapies, including ERT and SRT, that can cross the BBB [39,40], pharmacological chaperone therapy [41], and gene therapy [42] are under development as potential treatments for neurological symptoms. The usefulness of NBS for GD can be enhanced by the successful application of these treatments.

This study is limited in that NBS for GD was conducted in only a few regions of Japan. The population of Kumamoto and Fukuoka prefectures, where NBS was conducted, is only 5.4% of the Japanese population and does not reflect Japan as a whole. NBS for GD is now being initiated in many other regions of Japan, and the collective findings of these studies will provide more accurate patient numbers and variant frequencies.

## 5 Conclusions

We screened 155,442 newborns through NBS and diagnosed four newborns with GD in Japan. Three patients presented with signs or symptoms of neuronopathic GD. The frequency of GD diagnosis through NBS was 1 in 77,720, which was higher than that previously estimated. In the future, NBS for GD is expected to be performed in many regions of Japan and contribute to detecting more patients with GD. Early screening and diagnosis may have a very significant impact on the quality of life and potentially longevity in infants with GD.

## Funding

This study was supported in part by a Health and Labor Sciences Research Grant for Research on Rare and Intractable Diseases from the 10.13039/501100003478Ministry of Health, Labour and Welfare, Japan (grant number JPMH20FC1025); a Grant-in-Aid for Practical Research Project for Rare/Intractable Diseases from the 10.13039/100009619Japan Agency for Medical Research and Development (AMED; grant numbers JP19ek0109276, JP20ek0109482); and a Grant-in-Aid for Scientific Research from the Ministry of Education, Culture, Sports, Science, and Technology, Japan (10.13039/501100001691Japan Society for the Promotion of Science [JSPS] KAKENHI: grant number JP20K08207).

## Authors' contributions

TS, JK and KN were responsible for the design of the research. SY, SM, TS, YM and TI contributed to measurements and data collection. TS, JK, KS, SY, SH, FE and KN checked and analyzed the data. TS, JK and KS wrote the manuscript. JK and KN supervised this study. All authors read and approved the final manuscript for submission. All authors have agreed both to be personally accountable for the author's own contributions and to ensure that questions related to the accuracy or integrity of any part of the work, even ones in which the author was not personally involved, are appropriately investigated, resolved, and the resolution documented in the literature.

## Declaration of Competing Interest

All authors declare that there are no conflicts of interest associated with this study.

## Appendix A Supplementary data

Supplementary material 1 The location of *GBA*, *MTX1P1*, *GBAP1,* and *MTX1* and the target region of long-range PCR. A: Location of *GBA*, *MTX1P1*, *GBAP1* and *MTX1*. B: Structure of *GBA* gene and the target region of long-range PCR. C: Electrophoresis of long-range PCR product. The size of PCR products in all patients (Patient 1–4) were 13.6 kbp. Image 1

Supplementary material 2 Comparison of the *GBA* gene and *GBAP1* gene. Exons are depicted as boxes with numbers. Locations of recombination sites of the reported *GBA* recombinant alleles are indicated as Rec 1 to 8. The gap in the introns of *GBSP1* are indicated as dotted boxes with their length. Nucleotide substitutions and deletions are shown using arrows. Image 2
