# Supplementary material for: Newborn screening for Gaucher disease in Japan
Source: Mol Genet Metab Rep. 2022 Feb 18;31:100850. doi: 10.1016/j.ymgmr.2022.100850 (PMC8866142; doi:10.1016/j.ymgmr.2022.100850)
Supplement: Supplementary file 1 — Supplementary material 1 The location of GBA, MTX1P1, GBAP1, and MTX1 and the target region of long-range PCR. A: Location of GBA, MTX1P1, GBAP1 and MTX1. B: Structure of GBA gene and the target region of long-range PCR. C: Electrophoresis of long-range PCR product. The size of PCR products in all patients (Patient 1–4) were 13.6 kbp. [file mmc1.pptx]

## Slide 1
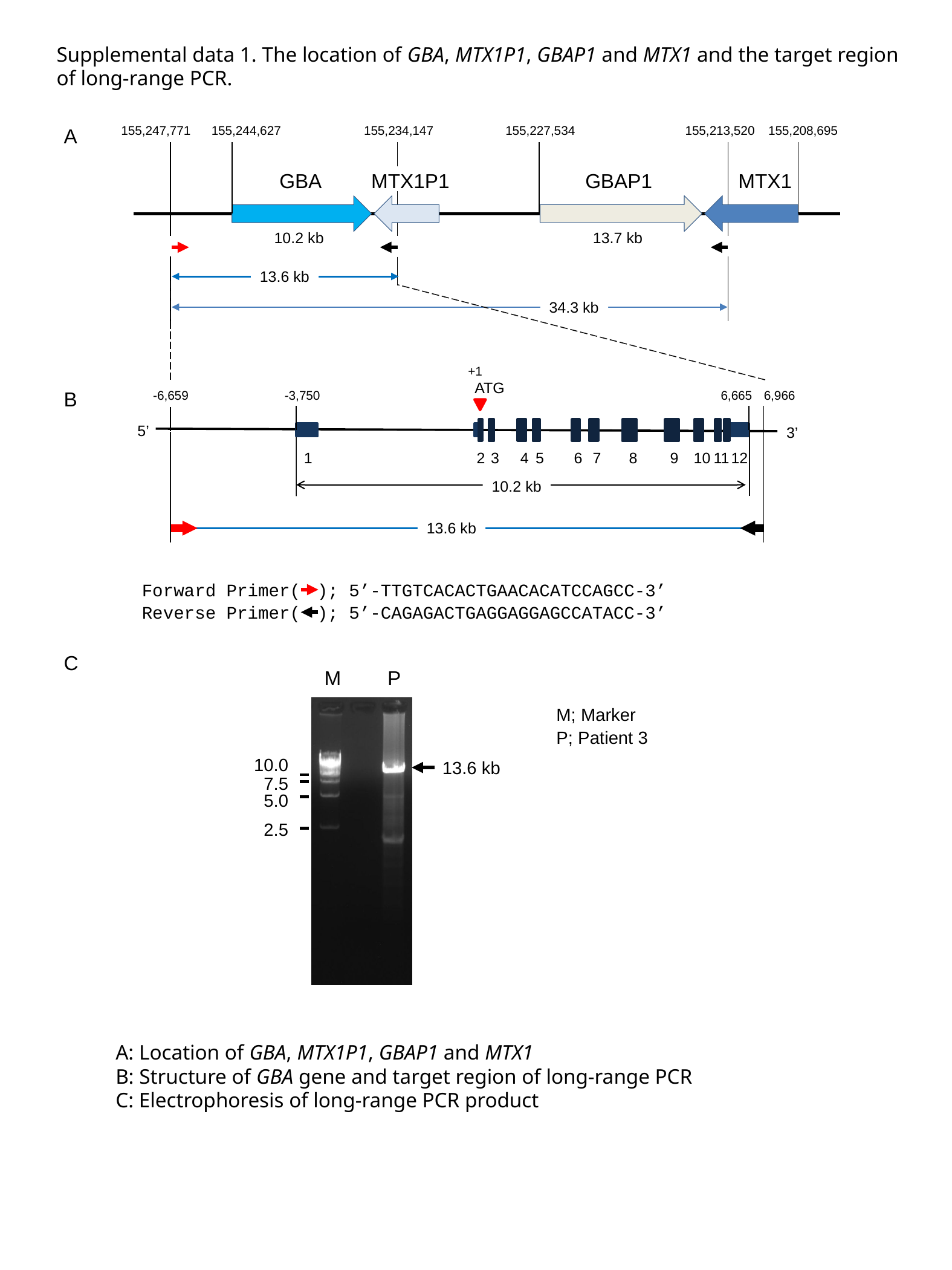

Supplemental data 1. The location of GBA, MTX1P1, GBAP1 and MTX1 and the target region of long-range PCR.
155,247,771
155,244,627
155,234,147
155,227,534
155,213,520
155,208,695
A
GBA
MTX1P1
GBAP1
MTX1
10.2 kb
13.7 kb
13.6 kb
34.3 kb
+1
ATG
B
-6,659
-3,750
6,665
6,966
5’
3’
1
2
3
4
5
6
7
8
9
10
11
12
10.2 kb
13.6 kb
Forward Primer( ); 5’-TTGTCACACTGAACACATCCAGCC-3’
Reverse Primer( ); 5’-CAGAGACTGAGGAGGAGCCATACC-3’
C
M
P
M; Marker
P; Patient 3
10.0
13.6 kb
7.5
5.0
2.5
A: Location of GBA, MTX1P1, GBAP1 and MTX1
B: Structure of GBA gene and target region of long-range PCR
C: Electrophoresis of long-range PCR product
