# Supplementary material for: Newborn screening for Gaucher disease in Japan
Source: Mol Genet Metab Rep. 2022 Feb 18;31:100850. doi: 10.1016/j.ymgmr.2022.100850 (PMC8866142; doi:10.1016/j.ymgmr.2022.100850)
Supplement: Supplementary file 2 — Supplementary material 2 Comparison of the GBA gene and GBAP1 gene. Exons are depicted as boxes with numbers. Locations of recombination sites of the reported GBA recombinant alleles are indicated as Rec 1 to 8. The gap in the introns of GBSP1 are indicated as dotted boxes with their length. Nucleotide substitutions and deletions are shown using arrows. [file mmc2.pptx]

## Slide 1
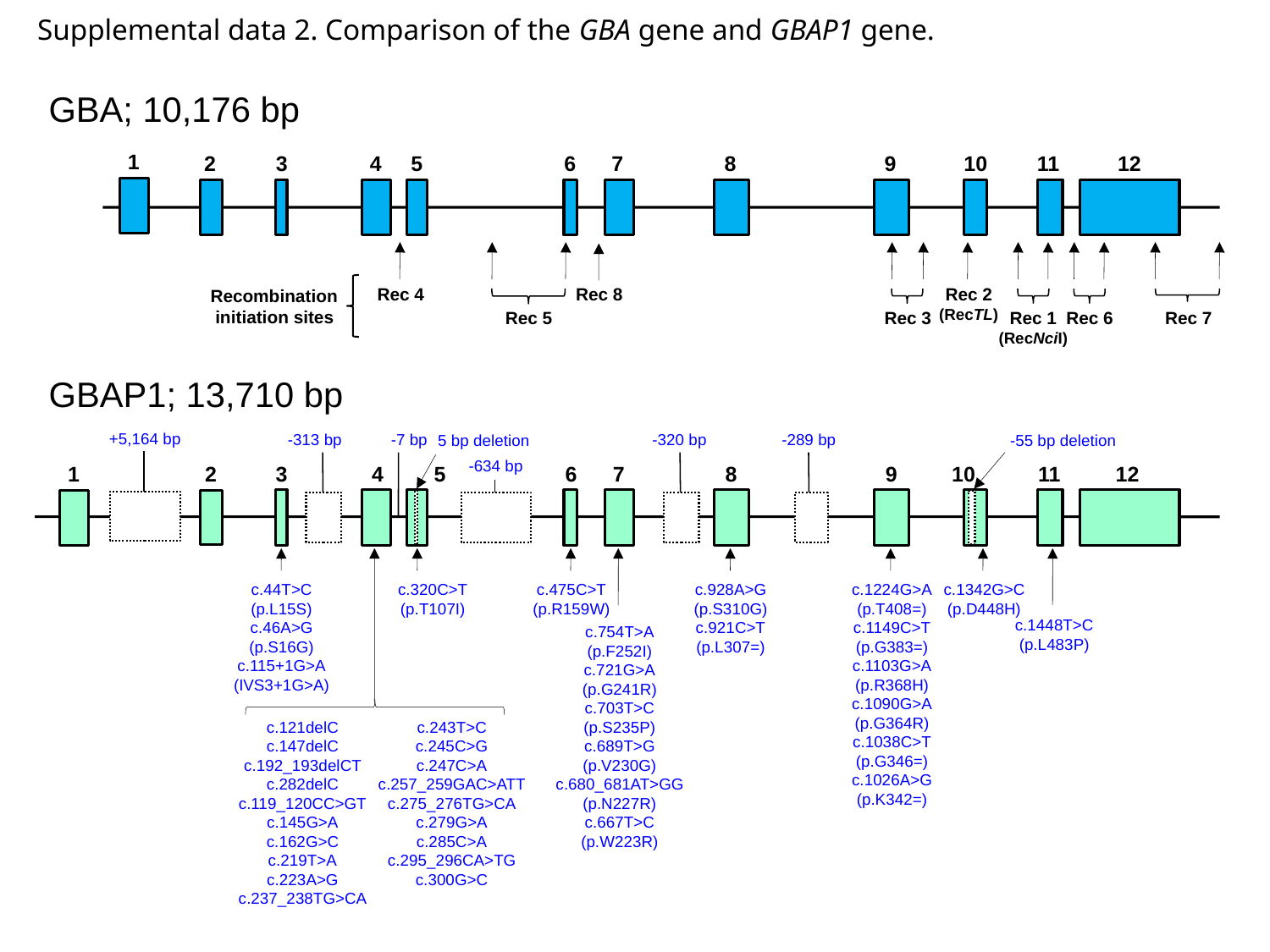

Supplemental data 2. Comparison of the GBA gene and GBAP1 gene.
GBA; 10,176 bp
1
2
3
4
5
6
7
8
9
10
11
12
Rec 4
Rec 8
Rec 2
(RecTL)
Recombination
 initiation sites
Rec 5
Rec 3
Rec 1
(RecNciI)
Rec 6
Rec 7
GBAP1; 13,710 bp
+5,164 bp
-313 bp
-7 bp
-320 bp
-289 bp
5 bp deletion
-55 bp deletion
-634 bp
1
2
3
4
5
6
7
8
9
10
11
12
c.44T>C
(p.L15S)
c.46A>G
(p.S16G)
c.115+1G>A
(IVS3+1G>A)
c.320C>T
(p.T107I)
c.475C>T
(p.R159W)
c.928A>G
(p.S310G)
c.921C>T
(p.L307=)
c.1224G>A
(p.T408=)
c.1149C>T
(p.G383=)
c.1103G>A
(p.R368H)
c.1090G>A
(p.G364R)
c.1038C>T
(p.G346=)
c.1026A>G
(p.K342=)
c.1342G>C
(p.D448H)
c.1448T>C
(p.L483P)
c.754T>A
(p.F252I)
c.721G>A
(p.G241R)
c.703T>C
(p.S235P)
c.689T>G
(p.V230G)
c.680_681AT>GG
(p.N227R)
c.667T>C
(p.W223R)
c.121delC
c.147delC
c.192_193delCT
c.282delC
c.119_120CC>GT
c.145G>A
c.162G>C
c.219T>A
c.223A>G
c.237_238TG>CA
c.243T>C
c.245C>G
c.247C>A
c.257_259GAC>ATT
c.275_276TG>CA
c.279G>A
c.285C>A
c.295_296CA>TG
c.300G>C
